# Simultaneous Beat-by-Beat Investigation of the Effects of the Valsalva Maneuver on Left and Right Ventricular Filling and the Possible Mechanism

**DOI:** 10.1371/journal.pone.0053917

**Published:** 2013-01-14

**Authors:** Zhen Wang, Li-jun Yuan, Tie-sheng Cao, Yong Yang, Yun-you Duan, Chang-yang Xing

**Affiliations:** Department of Ultrasound Diagnostics, Tangdu Hospital, Fourth Military Medical University, Xi’an, China; Idaho State University, United States of America

## Abstract

Although the influence of the Valsalva maneuver on the heart and circulatory system has been investigated, the mechanism of intrathoracic pressure influencing cardiovascular function is unclear. To test our hypothesis that the interaction between the anatomy-determined partially-intrathoracic system and the fully-intrathoracic system might explain those issues and help to disclose the mechanism, we used the Hitachi dual pulse wave Doppler echocardiographic apparatus to investigate simultaneously the beat-by-beat influence of 40-mmHg Valsalva maneuver on left and right cardiac ventricular filling in 30 male adult volunteers. The mitral and tricuspid blood inflow velocity spectra during the Valsalva maneuver were recorded simultaneously. The peak velocity (PV), velocity–time integral (VTI) and inflow volume (IV) of each cycle were measured or calculated. The PV, VTI and IV of the left heart remained unchanged at the first beat after the Valsalva maneuver onset (compared with those at rest, *p*>0.1) and then decreased gradually to the lowest at the 11±1.2th beat (range, 9^th^ to 12^th^ beat). Simultaneously, the PV, VTI and IV of the right heart decreased significantly (*p*<0.05) at the first cycle, decreased rapidly to the lowest at the 6±0.8th beat (range, 4^th^ to 7^th^ beat) and then increased gradually to the 9±1.3th beat (range, 8^th^ to 10^th^ beat). These results suggest that the left heart and right heart have different physiological responses to the Valsalva maneuver. These could be explained by our hypothesis, the interaction between the partially-intrathoracic system and the fully-intrathoracic system, which might help to disclose the mechanism of how intrathoracic pressure influences the heart and circulatory system.

## Introduction

The Valsalva maneuver (VM) has been used to raise intrathoracic pressure to investigate cardiovascular hemodynamic responses to changes in intrathoracic pressure in healthy volunteers and patients for many years [1]–[8]. The mechanism by which intrathoracic pressure influences cardiovascular function has always been of interest to physiologists and clinicians investigating circulatory disorders. However, exploration of the mechanism has been hindered because the effects of intrathoracic pressure on left and right ventricular function were previously determined separately, not simultaneously [9], [10]. In addition, the changes in each cardiac cycle during VM were incompletely investigated in previous beat-by-beat studies [11], [12], in which the early, mid and late phases of strain and recovery were divided. Consequently, the hemodynamic mechanism is unclear. To explore the mechanism, we hypothesized that the left and right cardiac responses should be investigated simultaneously and compared on a beat-by-beat basis.

However, measuring simultaneously the beat-by-beat left and right cardiac functional parameters has not been available because of technical problems. Presently, the dual pulse wave (PW/PW) Doppler echocardiographic technique developed by Hitachi (Tokyo, Japan) enables simultaneous recording and measurement of the relative parameters in two parts of cardiac blood flow in each cycle. This echocardiographic technique may be expected to be used to test and verify our proposed hypothesis on the mechanism of action. That is, the cardiovascular system can be divided into fully-intrathoracic and partially-intrathoracic enclosed fluid systems based on hydromechanics, and that their interactions may reflect and explain the underlying mechanism. Our proposed hypothesis is also expected to provide a further understanding on the nature of interactions between the respiratory and circulatory systems (e.g. Pulsus Paradoxus) which have puzzled us for so long time [13].

The purpose of the present study was to simultaneously explore and analyze the beat-by-beat changes in inflow volume and peak blood flow in left and right ventricular filling during the VM in normal subjects using dual PW/PW Doppler echocardiography and discuss the potential mechanism.

## Methods

### Ethical Approval of the Study Protocol

The study protocol was approved by the Ethics Committee of the Fourth Military Medical University (Xi’an, China). Written informed consent was obtained from each subject after the study was explained to him. All procedures were undertaken according to the Declaration of Helsinki.

### Subjects

Thirty male adult volunteers aged from 20 years to 45 years (mean age, 31±7.7 years) participated in this study. Subjects were normotensive, non-smoking, non-obese (body mass index (BMI) <30), and without cardiac or pulmonary abnormalities or other chronic diseases (determined by a general assessment based on medical history, physical examination and routine echocardiographic examination). All subjects were asked to refrain from consumption of tea, alcohol, or coffee for ≤24 h before the study commenced.

### Echocardiography

Echocardiography was undertaken in all subjects using an Hi Vision Preirus Ultrasonography system (Hitachi) with a 1–5-MHz transducer (EUP-S70). The dual PW/PW function of this system with two independent PW sample volumes (length was set at 3.5 mm) enabled us to record simultaneously mitral and tricuspid blood flow spectra at apical four-chamber views during the VM.

### Experimental Protocol

**Figure 1:**
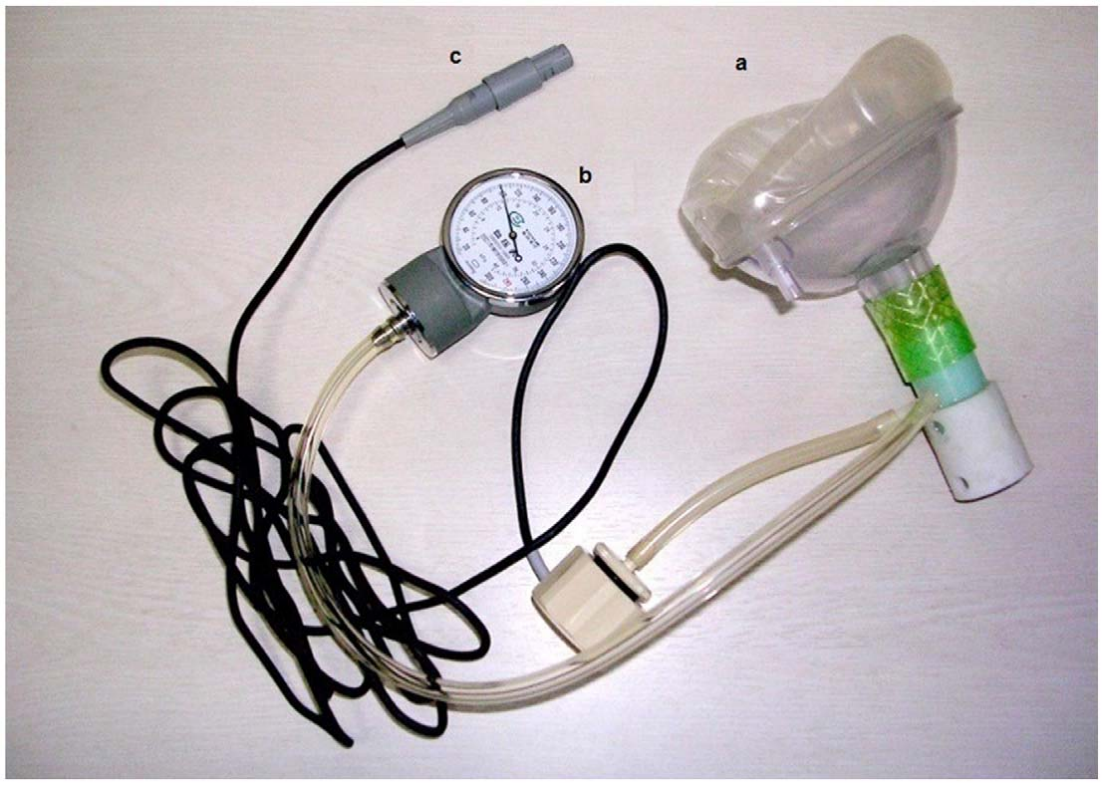
The pressure measurement device used in this study. The mask (**a**) with related tube was connected to the watch-shaped manometer (**b**), which was detached from an aneroid sphygmomanometer (XB-11 aneroid sphygmomanometer, Shanghai Medical Equipment Factory, Shanghai, China), and the needle of the manometer was set at 100 mmHg as the zero point. The pulse transducer (**c**) with wire and plug could be attached to the ultrasonographic system to display the action of the Valsalva maneuver.

A 40-mmHg VM was carried out by each subject with nose clips using a mask connected to a watch-shaped manometer. This manometer could read the positive or negative pressure, and had been calibrated using a standard mercurial sphygmomanometer according to a previous study [14]. Figure 1 demonstrates the pressure measurement device we used. The VM was maintained at the desired pressure (phases I and II) for 15 cardiac cycles (≈10 s), consistent with that employed in previous studies [1], [11], [15]. After the 15^th^ cycle, the release (phase III) began by giving the subject a sign, and the recording lasted to the 25^th^ cardiac cycle. All of the cardiac cycles were marked by an independent observer by counting the beat numbers from real-time echocardiography. All subjects were instructed to practice each maneuver several times to become accustomed to the procedure.

Each subject adopted the left oblique position at rest. The transducer was located at the point of the apical impulse or made a slight adjustment at this area to get a clear display of an apical four-chamber view of the heart, with good visualization of the left and right ventricular cavity with maximal excursion of the valve leaflets. Then, the two sample volumes were placed at the level of the mitral and tricuspid valve annulus, respectively, as guided by real-time two-dimensional (2D) imaging. We slightly adjusted the inception angle (all <20° in this investigation) if we needed to maximize inflow velocity and the graphic quality of the spectrum. After five stable cardiac cycles, the subject was asked to carry out the VM. Electrocardiography (ECG) was recorded simultaneously.

The peak velocity (PV) of each beat inflow (i.e., the E wave of inflow) through the mitral and tricuspid valves was measured on the PW/PW recording spectrum, and the velocity–time integral (VTI) of each beat was derived by tracing the velocity curve outline. The inflow volume (IV) of the left heart was calculated using the mitral inflow method by the equation:
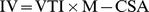
which has been validated by comparison with the thermodilution measurements in the work of Lewis et al. [16]. In this equation, VTI was obtained from the mitral inflow curve on the spectrum. The cross-sectional area of the mitral annulus (M-CSA) was calculated as π × (D/2)^2^, where D is the mid-diastolic transverse diameter of the mitral annulus obtained by measuring the inner edge of the lateral bright corner of the annulus to the inner edge of the medial corner just below the insertion of the mitral leaflets in clear 2D imaging at the apical four-chamber view. The cross-sectional area of the tricuspid annulus (T-CSA) was determined by dividing the tricuspid VTI (T-VTI) by IV derived from the mitral inflow method. The baseline values of M-CSA and T-CSA were determined as an average of five cardiac cycles at rest as well as with respiration hold. The beat-by-beat IV of the left or right heart during the VM was calculated by using the corresponding value of mitral VTI (M-VTI) or T-VTI and multiplying it by the baseline value of M-CSA or T-CSA.

### Intra- and Inter-observer Variability

Fifteen subjects were selected randomly to assess the intra-observer variability by the same observer as well as by another independent experienced observer to test the inter-observer variability. The repeated measurements of the cross-sectional area of the annuli, PV and VTI were undertaken in the same condition with an interval of one day. Observers were blinded to each other’s measurements. Inter- and intra-observer variability was calculated as the absolute difference between the corresponding repeated measurements divided by their mean, and expressed as a percentage.

### Statistical Analyses

Data are the mean ± SD. Results were analyzed using SPSS ver15.0 (SPSS, Chicago, IL, USA). Paired *t* tests (after the normal distribution test) were used to assess differences between two data groups. p<0.05 was considered significant.

## Results

### Characteristics of the Subjects

All subjects completed the experimental protocol. The baseline characteristics of the subjects are presented in Table 1. The values of the M-CSA and T-CSA of the subjects were 4.15±0.34 cm^2^ (D was 2.3±0.11 cm) and 4.93±0.39 cm^2^, respectively.

**Table 1:** The common characteristics of the 30 volunteers of the in vivo study.

	Results (n = 30)
**Age, yr**	31±7.7
**Height, cm**	174±10.2
**Weight, kg**	65.7±9.2
**Heart Rate, bpm**	69±4.6
**BMI, kg/m^2^**	24.8±3.7
**LAD, mm**	31±2.3
**LVEDD, mm**	46±5.8
**EF, %**	66±7.3

Values are means ± SD. BMI, Body Mass Index; LAD, Left Atrial Dimension; LVEDD, Left Ventricular End-Diastolic Dimension; EF, Ejection Fraction.

### Cardiac Beat-by-beat Responses to the VM

**Figure 2:**
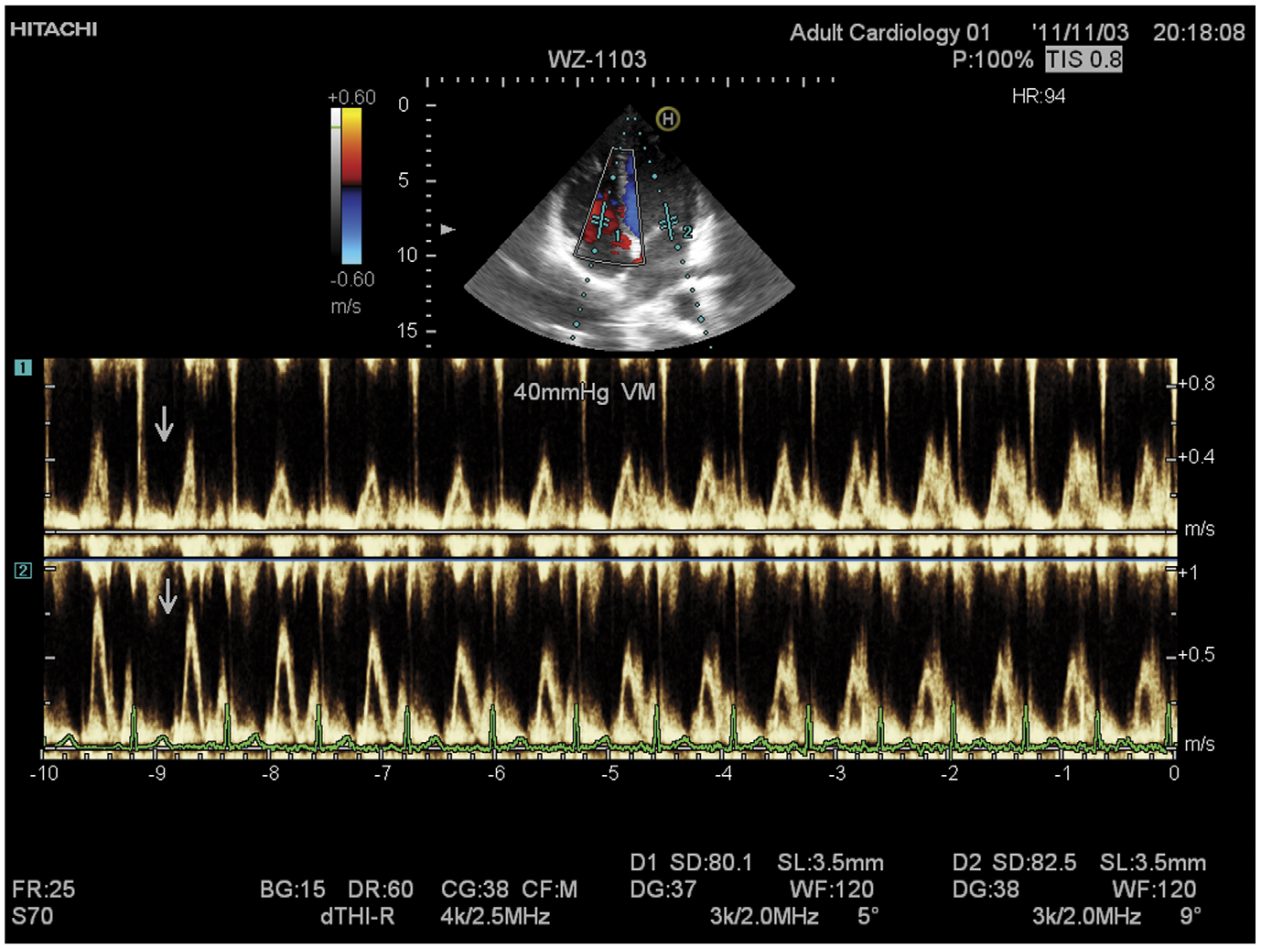
The beat-by-beat responses to Valsalva maneuver (VM) were simultaneously recorded by dual PW/PW echocardiography. The arrows show the beginning of a 40 mmHg VM. The tricuspid inflow spectrum (upper in each panel) and mitral inflow spectrum (lower in each panel) were simultaneously recorded. The peak velocity and the velocity-time integral (VTI) of each cardiac cycle can be measured in the spectrum. The bottom is the simultaneous ECG recording.

**Figure 3:**
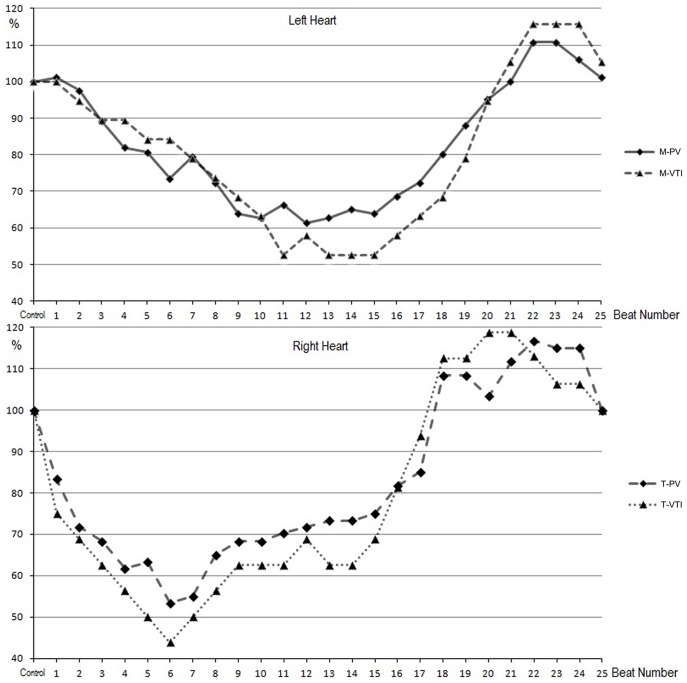
Average percent changes in peak velocity (PV) and velocity-time integral (VTI). The average percent of each beat was calculated as (mean value of each beat/the value at rest) ×100%. The upper panel shows the average percent changes of the peak inflow velocity of mitral valve (M-PV), the mitral VTI (M-VTI). The lower panel shows the average percent changes of the peak inflow velocity of tricuspid valve (T-PV), the tricuspid VTI (T-VTI).

**Figure 4:**
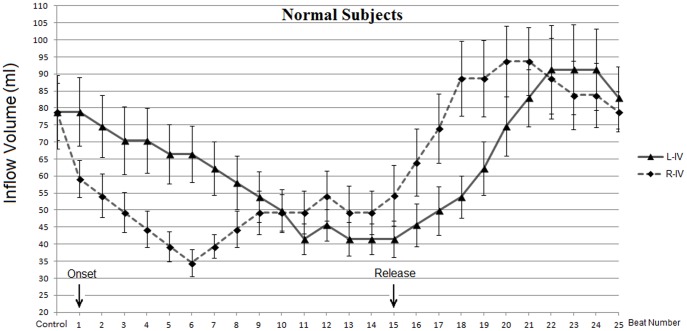
Changes in inflow volume of left heart (L-IV) and right heart (R-IV). The arrows below show the onset and the release of the 40 mmHg VM.

The beat-by-beat responses of the left and right heart to the 40-mmHg VM were recorded simultaneously by the dual PW/PW echocardiographic technique (Figure 2). The results of the right and left heart are illustrated graphically in Figure 3. For the left heart, The values of the PV, VTI and IV were unchanged at the first beat after the onset of the VM (compared with those at rest, *p = *0.13), began to decrease gradually, fell to the lowest value at the 11±1.2th beat (range, 9^th^ to 12^th^ beat) and then kept at this stable level until the release phase (phase III, began at the 16th beat) (Figure 3). Simultaneously, the values of the PV, VTI and IV of the right heart decreased significantly compared with those at rest (60±6.7 *vs* 50±5.8 cm/s; 16±1.7 *vs* 12±1.1 cm; 78.9±8.4 *vs* 59.2±5.4 ml; p<0.05) at the first cycle, then decreased rapidly to the lowest at 6±0.8th beat (range, 4^th^ to 7^th^ beat), and then increased gradually to the 9±1.3th beat (range, 8^th^ to 10^th^ beat) and kept at this level until the release phase (Figure 3). After the release (from the 16th beat), the values of the PV, VTI and IV of the right and left heart increased, and an overshoot was observed. On average, the IV of the left heart reached the peak at the 22nd beat and increased by 15.8% compared with that at rest. The IV of the right heart increased to the maximum at the 20th beat and increased by 18.8%. Figure 4 compares, in a graphical format, the changes in IV of the left heart and right heart during and after the VM. Right-heart IV was lower than of the left heart before the 10±1.6th (range, 8^th^ to 11^th^) beat, and then was higher than the left IV until almost the 22nd beat (between the 21^st^ and 23^rd^ beat). The changes of IV in the right heart were before those of the left heart during and after 40-mmHg VM.

### Inter- and Intra-observer Variations

The inter- and intra-observer variations in the measurements are shown in Table 2. The measurements of PV and VTI of 25 cycles had lower variations than the baseline measurements of M-CSA and T-CSA, which had the highest inter-observer variation (Table 2).

**Table 2:** Inter- and intraobserver variabilities in the measurements in fifteen subjects.

Variabilities	M-CSA	T-CSA	M-PV	T-PV	M-VTI	T-VTI
**Intra-observer variability, %**	11.4±6.6	12.7±6.8	3.3±0.7–6.0±1.2	3.6±0.8–7.2±1.3	4.5±1.0–8.8±2.3	5.3±2.6–9.6±2.8
**Inter-observer variability, %**	13.3±7.5	14.4±9.6	3.4±0.7–6.7±1.7	3.7±0.8–7.8±1.9	5.2±1.4–9.7±2.8	6.2±2.7–10.8±3.2

Values are means ± SD. M-CSA, the cross-sectional area of the mitral annulus; T-CSA, the cross-sectional area of the tricuspid annulus; M-PV, peak inflow velocity of mitral valve of 25 cycles; T-PV, peak inflow velocity of tricuspid valve of 25 cycles; M-VTI, mitral velocity-time integral of 25 cycles; T-VTI, tricuspid velocity-time integral of 25 cycles.

## Discussion

This present study investigated simultaneously the beat-by-beat influences of 40-mm Hg VM on left and right cardiac ventricular filling in 30 male adult subjects using a dual PW/PW echocardiographic technique. The results showed that the right heart and left heart had different physiological responses with regard to the PV, VTI and IV during the VM. This was the first study to investigate these issues non-invasively, simultaneously as well as on a beat-by-beat basis. This could be a new method to further explore the mechanism of heart-lung interactions.

The VM was firstly described by Valsalva in 1704. Since then, it has been employed frequently to mark changes such as pressure, cardiac-chamber size and single ventricular stroke volume during the VM [1], [3], [10], [17]–[21]. However, the underlying mechanism of action remains unclear. In the present study, significantly different responses to the VM between the left and right heart were noticed, which confirmed our theoretical expectations. Not only the findings of the present study, but also the results of other studies, could be explained by our proposed hypothesis.

**Figure 5:**
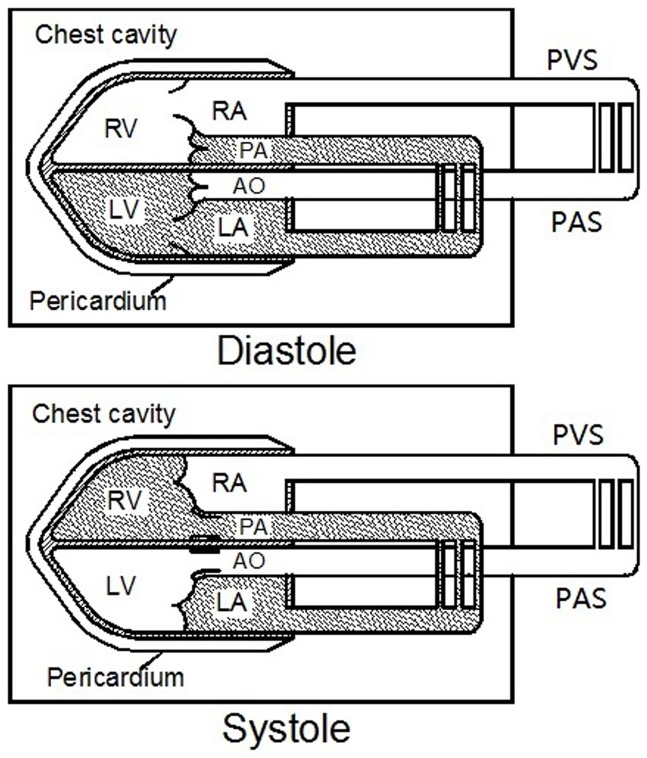
The fully-intrathoracic system and the partially-intrathoracic system of our hypothesis. Anatomically and functionally, the circulatory system can be divided into two parts, the fully-intrathoracic system (the dark part) is completely in the chest cavity, while the partially-intrathoracic system (the white part) is partly in the chest cavity and is connected with the vein or artery system outside the chest cavity. These two systems are a little different in diastole and in systole. RA = Right Atrium; RV = Right Ventricle; LA =  Left Atrium; LV = Left Ventricle; PA = Pulmonary Artery; AO = Aorta; PVS = Peripheral Venous System; PAS = Peripheral Arterial System.

The heart is confined to the chest cavity but is connected functionally to the outside through an artery and vein system by absorbing venous return through the tricuspid valve in diastole and ejecting blood through the aortic valve in systole. Figure 5 illustrates our hypothesis, which is based on hemodynamics and mechanics. During cardiac diastole, physiologically, the pulmonary valve and aortic valve are closed, and then the pulmonary artery (PA), lung, left atrium (LA) and left ventricle (LV) form a hydro-mechanically enclosed system in the chest cavity: here we name it as the “fully-intrathoracic system” (the dark part of the upper panel in Figure 5). At the same phase, the right atrium (RA), right ventricle (RV), the aorta (AO) and the thoracic aorta form a system which is connected with the vein or artery system outside the chest cavity, which we name here the “partially-intrathoracic system” (the white part of the upper panel in Figure 5). Similarly, during systole, the RV, PA, lung and LA are the fully-intrathoracic system in the chest cavity (the dark part of the lower panel in Figure 5) and the RA, LV, AO and the thoracic aorta are the partially-intrathoracic system (the white part of the lower panel in Figure 5). Based on Pascal’s law, changes in intrathoracic pressure will be transmitted without loss in any part of the enclosed fully-intrathoracic system on the assumption of its incompressibility. However, the pressure increasing in the partially-intrathoracic system is limited and slow because of the decreased systemic venous return caused by the VM. Furthermore, the RV is part of the partially-intrathoracic system at diastole and then shifts to be the part of the fully-intrathoracic system at systole. Meanwhile, the LV has a contrary transformation from diastole to systole. The shift of the roles of the RV and LV indicates that the fully-intrathoracic and partially-intrathoracic systems within the chest cavity communicate once in each cardiac cycle. Thus, the changes in intrathoracic pressure may directly and indirectly influence the function of the right heart and the left heart, and the influence might be different in time or extent.

When the VM was carried out, the intrathoracic pressure increased rapidly and was transmitted into the heart and other tissues within the chest cavity. At the diastole of the first cycle, for the left heart, the LA and LV belong to the fully-intrathoracic system (Figure 5). The increase in pressure is transmitted to the entire enclosed system, and no pressure difference is produced between the LA and LV, thus the PV, M-VTI and IV remained unchanged. Simultaneously, for the right heart, the RA and RV (partially-intrathoracic system) are connected to the peripheral vein system outside the cavity by the vena cava. The increase in intrathoracic pressure directly impeded venous return to RV and diastolic filling decreased, so the PV, T-VTI and IV decreased. When in systole, the tricuspid valve closes, and the RV become part of fully-intrathoracic system, and the decreased IV was ejected into the PA and lung, resulting in a pressure decrease in the fully-intrathoracic system (including the LA). In the next cardiac cycle, the depressed pressure in the PA, lung and LA decreased the diastolic filling of the LV, so the PV, M-VTI and IV of the left heart began to decrease gradually. During the strain phase of the VM, as venous return was impeded, the peripheral venous pressure increased because a large amount of blood was accumulatively pooled into the periphery [2], [10], and diastolic filling of the RV increased gradually after its lowest point to reach a stable level to balance the increased intrathoracic pressure caused by the VM (Figures 2, 3, 4). Hence, when the VM released, an overshoot of the right and then left was detected (Figure 4) because the partially-intrathoracic system had a higher pressure level due to the accumulation of blood which increased filling of the RV and then of the LV.

Studies [9], [22] have shown that subjects with congestive heart failure had different responses during the Valsalva strain. That is, despite the significant decrease in systemic venous return and stroke volume of the right heart, the filling and stroke volume of the left heart was maintained and unchanged. Those findings could also be explained by our hypothesis. In those patients, the enclosed system including the PA, the lung and LA was at a high level of pressure because of congestion, which might result in an elevated pressure in the fully-intrathoracic system. Thus, although the decreased volume of the right heart was ejected discontinuously into the enclosed system, the fully-intrathoracic system acted as a kind of “reservoir” that could maintain its pressure because the decreased right-heart volume was too limited to influence the pressure. This might be why changes and overshoot were not observed in those patients.

According to our hypothesis, at the beginning of the VM (especially in the first cardiac cycle), the pressure difference between the interventricular septum increases because the RV (partially-intrathoracic system) has a limited increase in pressure due to decreased venous return whereas the pressure in the LV (fully-intrathoracic system) increases equally by the VM according to Pascal’s law. As a result, this increased pressure difference may push the interventricular septum shift rightward, which may theoretically increase filling of the LV. However, the interventricular septum has already shifted rightward at rest and will have a certain tension because the diastolic pressure in the LV is higher at physiological conditions. This increase in LV filling is limited and might be dependent upon the individual, so it does not cause hemodynamic changes in the left heart, which was also confirmed by another study [9]. However, during cardiac tamponade or the Mueller maneuver, septal displacement would be significant [23], [24], which could also be explained using our hypothesis. Furthermore, our hypothesis might be expected to provide further understanding of the cardiac hemodynamic changes not only in physiological behaviors such as cough and bowel movements, but also in pathological states such as obstructive sleep apnea, congestive heart failure and pericardial tamponade.

The limitation of the present study was that, although the accuracy and practicality of the mitral inflow method has been confirmed by Lewis et al. [16], the assumption that the cross-sectional area of the valvular annulus remained unchanged during the VM could have influenced the accuracy of the calculation of the IV. The mitral and tricuspid annuli are seated at the fibrous ring of the cardiac floor and fibrous valvular tissues have poor flexibility. Hence, we infer that the influence of the VM on valvular cross-sectional area may be limited.

### Conclusions

The results of this simultaneous beat-by-beat study suggested that the left heart and right heart have different physiological responses to cardiac filling during the VM in humans. Our proposed hypothesis (i.e., the interaction between the partially-intrathoracic system and the fully-intrathoracic system) might help to explain the mechanism of intrathoracic pressure influencing the heart and circulatory system.

## References

[1] Sharpey-Schafer EP (1955) Effects of Valsalva's manoeuvre on the normal and failing circulation. Br Med J 1: 693–695. 14351748 10.1136/bmj.1.4915.693 PMC2061457

[2] Porth CJ, Bamrah VS, Tristani FE, Smith JJ (1984) The Valsalva maneuver: mechanisms and clinical implications. Heart Lung 13: 507–518. 6565684

[3] Buda AJ, Pinsky MR, Ingels NB, Daughters GT, Stinson EB, et al (1979) Effect of intrathoracic pressure on left ventricular performance. N Engl J Med 301: 453–459. 460363 10.1056/NEJM197908303010901

[4] Aliverti A, Bovio D, Fullin I, Dellacà RL, Lo Mauro A, et al (2009) The Abdominal Circulatory Pump. PLoS ONE 4(5): e5550 doi:10.1371/journal.pone.0005550. 19440240 10.1371/journal.pone.0005550 PMC2678249

[5] Monge Garcı'a MI, Gil Cano A, Dı´az Monrove JC (2009) Arterial pressure changes during the Valsalva maneuver to predict fluid responsiveness in spontaneously breathing patients. Intensive Care Med 35: 77–84. 18830578 10.1007/s00134-008-1295-1

[6] Oh JK, Park SJ, Nagueh SF (2011) Established and novel clinical applications of diastolic function assessment by echocardiography. Circ Cardiovasc Imaging 4: 444–455. 21772012 10.1161/CIRCIMAGING.110.961623

[7] Silber HA, Trost JC, Johnston PV, Maughan WL, Wang NY, et al (2012) Finger photoplethysmography during the Valsalva maneuver reflects left ventricular filling pressure. Am J Physiol Heart Circ Physiol 302: H2043–H2047. 22389389 10.1152/ajpheart.00609.2011 PMC3774124

[8] Stewart JM, Medow MA, Bassett B, Montgomery LD (2004) Effects of thoracic blood volume on Valsalva maneuver. Am J Physiol Heart Circ Physiol 287: H798–H804. 15059782 10.1152/ajpheart.01174.2003

[9] Little WC, Barr WK, Crawford MH (1985) Altered effect of the Valsalva maneuver on left ventricular volume in patients with cardiomyopathy. Circulation 71: 227–233. 3965168 10.1161/01.cir.71.2.227

[10] Robertson D, Stevens RM, Friesinger GC, Oates JA (1977) The effect of the Valsalva maneuver on echocardiographic dimensions in man. Circulation 55: 596–602. 837502 10.1161/01.cir.55.4.596

[11] Greenfield JC, Cox RL, Hernandez RR, Thomas C, Schoonmaker FW (1967) Pressure-flow studies in man during the Valsalva maneuver with observations on the mechanical properties of the ascending aorta. Circulation 35: 653–661. 5337274 10.1161/01.cir.35.4.653

[12] Parisi AF, Harrington JJ, Askenazi J, Pratt RC, McIntyre KM (1976) Echocardiographic evaluation of the Valsalva maneuver in healthy subjects and patients with and without heart failure. Circulation 54: 921–927. 991407 10.1161/01.cir.54.6.921

[13] Cannesson M, Aboy M, Hofer CK, Rehman M (2011) Pulse pressure variation: where are we today? J Clin Monit Comput 25: 45–56. 20390324 10.1007/s10877-010-9229-1

[14] Yuan LJ, Cao TS, Duan YY, Yang GD, Wang ZJ, et al (2004) Noninvasive assessment of influence of resistant respiration on blood flow velocities across the cardiac valves in humans–a quantification study by echocardiography. Echocardiography 21: 391–398. 15209717 10.1111/j.0742-2822.2004.03086.x

[15] McKay RG, Spears JR, Aroesty JM, Baim DS, Royal HD, et al (1984) Instantaneous measurement of left and right ventricular stroke volume and pressure-volume relationships with an impedance catheter. Circulation 69: 703–710. 6697458 10.1161/01.cir.69.4.703

[16] Lewis JF, Kuo LC, Nelson JG, Limacher MC, Quinones MA (1984) Pulsed Doppler echocardiographic determination of stroke volume and cardiac output: clinical validation of two new methods using the apical window. Circulation 70: 425–431. 6744546 10.1161/01.cir.70.3.425

[17] Brooker JZ, Alderman EL, Harrison DC (1974) Alterations in left ventricular volumes induced by Valsalva manoeuvre. Br Heart J 36: 713–718. 4411920 10.1136/hrt.36.7.713 PMC458885

[18] Gindea AJ, Slater J, Kronzon I (1990) Doppler echocardiographic flow velocity measurements in the superior vena cava during the Valsalva maneuver in normal subjects. Am J Cardiol 65: 1387–1391. 2343828 10.1016/0002-9149(90)91333-2

[19] Pinsky MR, Summer WR, Wise RA, Permutt S, Bromberger-Barnea B (1983) Augmentation of cardiac function by elevation of intrathoracic pressure. J Appl Physiol 54: 950–955. 6853301 10.1152/jappl.1983.54.4.950

[20] Robotham JL, Lixfeld W, Holland L, MacGregor D, Bryan AC, et al (1978) Effects of respiration on cardiac performance. J Appl Physiol 44: 703–709. 649472 10.1152/jappl.1978.44.5.703

[21] Sharpey-Schafer EP (1965) Effect of respiratory acts on the circulation. In: Handbook of physiology, edited by Hamilton WF, Dow P. Washington, DC: American Physiological Society.

[22] Hamilton WF, Woodbury RA, Harper HT Jr (1944) Arterial cerebrospinal and venous pressures in man during cough and strain. Am J Physiol 141: 42–50.

[23] Brinker JA, Weiss JL, Lappe DL, Rabson JL, Summer WR, et al (1980) Leftward septal displacement during right ventricular loading in man. Circulation 61: 626–633. 7353253 10.1161/01.cir.61.3.626

[24] Guzman PA, Maughan WL, Yin FC, Eaton LW, Brinker JA, et al (1981) Transseptal pressure gradient with leftward septal displacement during the Mueller manoeuvre in man. Br Heart J 46: 657. 7317234 10.1136/hrt.46.6.657 PMC482713

